# Surveying the Effect of Opioid Abuse on the Extent of Coronary Artery Diseases in Diabetic Patients

**DOI:** 10.1155/2020/8619805

**Published:** 2020-07-19

**Authors:** Seyyed Ali Moezi Bady, Maryam Soltani, Toba Kazemi, Saeede Khosravi Bizhaem, Nazanin Hanafi Bojd, Neda Partovi, Hamid Reza Mashreghimoghadam, Fatemeh Hamidi, Nahid Azdaki

**Affiliations:** ^1^Cardiovascular Diseases Research Center, Department of Cardiology, School of Medicine, Birjand University of Medical Sciences, Birjand, Iran; ^2^Razi Clinical Research Development Unit (RCRDU), Birjand University of Medical Sciences, Birjand, Iran

## Abstract

**Background:**

Diabetes mellitus is recognized as one of the most common, serious, and costly chronic diseases. Opium addiction is also a common health problem in Iran. Given the high prevalence of opium use in South Khorasan Province and the increasing prevalence of opioid abuse in the community, this study was performed to investigate the effect of opioid abuse on the extent of disease in diabetic patients undergoing coronary angiography in the cardiology department of Vali-e-Asr Hospital in Birjand city, South Khorasan Province, Iran.

**Methods:**

This study recruited a total of 1051 diabetic patients who underwent coronary angiography in the cardiology department of Vali-e-Asr Hospital of Birjand city from 2011 to 2015. The collected data were analyzed using SPSS version 22.0 with the chi-square test and univariate regression analysis. *P* value <0.05 was considered as statistically significant.

**Results:**

Among opiate-addicted diabetics, the risk of coronary artery disease was 0.44 times higher than among nonaddicted diabetics (range 0.24–0.77, *P*=0.004). The extent of coronary vessel involvement, when present, was not different between the two groups.

**Conclusion:**

Opiate-addicted diabetics appear to be more susceptible to CAD than their nonaddicted counterparts. The determinants and correlates of this interaction must be the subject of further study.

## 1. Introduction

Diabetes is a common, serious, and costly chronic disease in Iran [1]. Diabetic patients are subject to multiple complications such as blindness; kidney failure; foot ulcers; and large vessel disease such as coronary vessel involvement, stroke, and peripheral vascular disease leading to ischemia and lameness [2]. According to the recent statistics from the World Health Organization, more than 75% of the patients with type 2 diabetes die due to vascular disease [3]. Opium use is one of the most common health problems in Iran. In some regions of the world, especially in Asia and the Middle East, the use of opium has a protective effect on the control of CVDs, hypertension, and diabetes [4–6], which has driven higher rates of opium use in these areas. The prevalence of opium use is high in Iran, as 3% of the population is addicted to opium [6]. A number of studies have shown that opium usage can reduce some of the modifiable risk factors for CADs, whereas some studies showed harmful and hazardous effects of opium addiction on CADs [4, 7–9]. Marmer et al. stated that long-term opiate addiction was associated with a reduced extent of coronary vessel involvement and related fatal outcomes in autopsy samples [8]. However, another study reported that opium use may contribute to the development of atherosclerotic plaques [4]. Given the relatively high prevalence of opium use in South Khorasan Province and the increasing trend of opioid addiction in these areas, the present study was aimed to investigate the effect of opioid abuse on the extent of coronary artery disease in diabetic patients undergoing coronary angiography in the cardiology department of Vali-e-Asr Hospital in Birjand city.

## 2. Methods

This study was conducted in 1051 diabetic patients who underwent coronary angiography in the cardiology department of Vali-e-Asr Hospital of Birjand city from 2011 to 2015. The required data were collected using a researcher-made checklist. Accordingly, this checklist evaluated some variables such as age (e.g., <45, 45–65, and >65 years old), gender (male/female), family history of CVD (yes/no), opium use (yes/no), dyslipidemia (yes/no), history of hypertension (yes/no), body mass index (underweight/normal/overweight/obese), diabetes (yes/no), and extent of coronary vessel involvement. Then, the subjects were assigned into three groups based on the extent of coronary vessel involvement as follows: the normal CAD group comprised of patients without any coronary involvement on angiography, the no significant CAD group comprised of patients with coronary involvement less than 50%, and the significant CAD group comprised of patients with coronary involvement more than 50%. A diabetic patient is defined as a person with glucose level greater than 126 mg/dl or a history of diabetes medication [10]. Data were analyzed using SPSS version 22.0 with the chi-square test and univariate and multivariate regression analysis. Also, *P* value <0.05 was considered as statistically significant.

## 3. Results

Demographic and clinical characteristics of the participants are presented in Table 1. The findings of this study, between opiate-addicted and nonaddicted participants, respectively, are as follows: males: 56.1% vs. 45.7% (*P*=0.034); BMI >25: 51.9% vs. 63.8% (*P*=0.037); coronary vessel involvement >50%: 68.6% vs. 77.8% (*P*=0.013). No other significant demographic differences were observed between the two groups. Female opiate-addicted participants were 1.81 times more likely to have coronary vessel involvement (range: 1.03–3.15, *P*=0.037), with coronary artery disease 0.59 times higher (range: 0.35–0.98, *P*=0.045). No difference was observed between female opiate-addicted and nonaddicted female diabetics (OR: 1.06; range: 0.70–1.62, *P*=0.753). Among males, opiate addiction was not a correlate of coronary vessel involvement (OR: 1.09, range: 0.63–1.88, *P*=0.753) or coronary artery disease (Table 2). Among opiate-addicted diabetics, the risk of coronary artery disease was 0.44 times higher than among nonaddicted diabetics (range: 0.24–0.77, *P*=0.004). No other differences were statistically significant (Table 3).

## 4. Discussion

This study was aimed to examine the relationship between opium usage and extend of coronary vessel involvement among the diabetic patients. The incorrect beliefs on the beneficial effects of opium on life span and its protective effects against cardiac problems and heart attack play a significant role in the increased tendency of patients to use opium [11]. The findings of our study suggest that the odds of no significant were significantly higher in female opiate-addicted patients in normal and no significant groups. Also, the odds of CAD were shown to be significantly lower in female opiate-addicted patients than in opiate nonaddicted females in CAD and no significant groups. However, the risk of CAD was not significantly different between female opiate-addicted and opiate nonaddicted patients in normal and CAD groups. Moreover, comparing male opiate-addicted with male opiate nonaddicted patients in all the study groups (e.g., the normal group vs. the CAD group, the normal group vs. the no significant group, and the CAD group vs. the no significant group) revealed that the extend of coronary vessel involvement was not significantly different between male opiate-addicted and opiate nonaddicted patients. These results were inconsistent with the results of many studies, as Sadeghian et al. stated that opium use among men is the most important risk factor for CAD [12]. Also, Khodneva et al. reported higher risk of CHD in opiate-addicted female compared to opiate nonaddicted females [13]. A retrospective study performed by Sharafi et al. on 1,545 men undergoing PCI showed that vascular involvement was higher in male nonopioid users [14].

According to the literature, predisposing factors for CAD in opiate-addicted patients are hormonal changes such as changes in testosterone and estrogen levels, as well as insulin resistance. The reduced levels of testosterone in men are also associated with CAD and mortality due to cardiovascular disease [15]. Also, the decreased estrogen plasma levels in women during the premenopausal period can lead to the increased risk of CAD and consequently to death due to cardiovascular disease [16]. According to previous studies, higher rates of coronary artery involvement in opium users include (1) plasma testosterone levels, as coronary artery involvement was shown to be higher in men with low testosterone levels, (2) poor glucose monitoring leading to progression of CADs in opium user patients, and (3) long-term use of opium [7, 17–19]. The duration and amount of opiate use were not precisely determined, and hormonal levels of patients were not measured in this study, as well as lack of previous studies on the severity of coronary involvement among male and female diabetic patients with opium use; thus, the interpretation of results is limited. In addition, a cross-sectional design limits us to derive a causal relationship between opium use and the severity and rate of vascular involvement.

According to the results, the risk of CAD was shown to be significantly lower in opiate-addicted diabetics in the CAD group in comparison with the no significant group. However, the odds of coronary vessel involvement were not significantly different between opiate-addicted and opiate nonaddicted diabetics in the normal group compared to CAD and no significant groups. There are many studies with contrary results performed on the effect of opium addiction on risk factors associated with CVDs. As such, some of them proposed the protective effect of opium against CVDs and protect heart against ischemia reperfusion (IR) injury [20, 21], while this protective effect was not approved by many studies using coronary angiography [6, 22]. Also, a number of studies conducted on animals have shown the increased risk of atheroma plaque growth in the presence of opium [4]. A number of previous studies have shown that opioids reduce inflammation and CADs [8, 23] and suggested that the long-term use of opium may directly reduce atherosclerosis and protect people against ischemic injury and infraction [8]. Another study on patients with acute myocardial infraction (MI) found that sudden anterior wall MI was less common among opium users [24]. Other studies in Iran have suggested that opium may have a protective effect on cardiovascular risk factors, including diabetes, hypertension, and hyperlipidemia, as well as cardiovascular disease [4, 19, 25, 26].

The long-term use of opium is associated with the increased levels of many substances including substance P, calcitonin, adenosine, and adenylyl cyclase. These substances have shown to have protective effects on heart performance.

However, opium has various effects on the stimulation of kappa receptor, and the results show that kappa-opioid receptor stimulation can result in myocardial perfusion defects and increased myocardial infarct size [27, 28]. Marmor et al. proposed that the severity of CAD and prevalence of fatal myocardial infraction were lower among those individuals with opium addiction or methadone use. They also stated that long-term exposure to opiate drugs is associated with the reduced severity of CADs and related adverse complications [8]. Also, Hosseini et al. found that the scores of Gensini and coronary vessel involvement were higher in opiate-addicted diabetics compared to opiate nonaddicted diabetics [3]. Sadeghian et al. also demonstrated that the risk of CAD was higher in opiate-addicted patients compared with other patients [7]. Hosseini and Salehi conducted a study on patients who had undergone coronary angiography and found that a number of risk factors such as old age, high blood glucose level, male gender, waist-hip ratio, and opium use were significantly higher in CAD patients than in non-CAD patients [29]. Moreover, Masoomi et al. revealed that opium consumption is a risk factor for CAD [6], which was inconsistent with the results of our study. Azimzadeh et al. indicated no association between the history of opium use and myocardial infraction [30]. Roohafza et al. stated that opium usage was not associated with the increased risk of CAD rate and the increased death rate due to myocardial infraction [31]. Azod et al. also reported that opium use had no beneficial effects on blood glucose level among the diabetic patients [32]. In general, contradiction in the results of these studies can be attributed to the differences in their sample sizes, types of opiate use (e.g., oral or inhaled), types of consumed opioids, purity, and amount and duration of exposure to opioids [7, 8].

## 5. Conclusion

Opiate-addicted diabetics appear to be more susceptible to CAD than their nonaddicted counterparts. The determinants and correlates of this interaction must be the subject of further study.

## 6. Limitations

This was the first study performed on the relationship between opium addiction and extend of coronary vessel involvement with a large sample size in South Khorasan Province. However, there was only one item in our checklist to identify opiate-addicted patients, and the dose and duration of opium use were not evaluated in our study; therefore, the number of patients with opioid addiction was underestimated in our study. Hence, the validity and accuracy of the results were not approved. Besides, self-report bias due to public stigma on opiate addiction reduced the precision of results.

## Figures and Tables

**Table 1:** Demographics and clinical characteristics of the participants.

Characteristics	Opiate-addicted diabetics, *N* (%), 123 (11.7)	Nonaddicted diabetics, *N* (%), 928 (88.3)	Total *N* = 1051	*P* value
Age (years)				
45>	7 (5.9)	54 (5.9)	61 (5.9)	0.905
45–65	75 (63.6)	564 (61.6)	639 (61.8)	
65<	36 (30.5)	298 (32.5)	334 (32.3)	
Sex (%)				
Female	54 (43.9)	504 (54.3)	558 (53.1)	0.034
Male	69 (56.1)	424 (45.7)	493 (46.9)	
Family history of CVD (%)				
No	107 (87)	833 (89.8)	940 (89.4)	0.349
Yes	16 (13)	95 (10.2)	111 (10.6)	
Hypertension (%)				
No	58 (47.2)	449 (48.4)	507 (48.2)	0.848
Yes	65 (52.8)	479 (51.6)	544 (51.8)	
Dyslipidemia (%)				
No	57 (46.3)	429 (46.2)	486 (46.2)	1
Yes	66 (53.7)	499 (53.8)	565 (53.8)	
BMI (%)				
<18.5 underweight	4 (3.7)	17 (2.2)	21 (2.4)	0.037
18.5–24.99 normal	48 (44.4)	263 (34)	311 (35.3)	
>25 overweight and obese	56 (51.9)	494 (63.8)	550 (62.4)	
Single-vessel disease (%)				
No	109 (88.6)	778 (83.8)	887 (84.4)	0.170
Yes	14 (11.4)	150 (16.2)	164 (15.6)	
Two-vessel disease (%)				
No	97 (78.9)	780 (84.1)	877 (83.4)	0.146
Yes	26 (21.1)	148 (15.9)	174 (16.6)	
Three-vessel disease (%)				
No	86 (69.9)	567 (61.1)	653 (62.1)	0.058
Yes	37 (30.1)	361 (38.9)	398 (37.9)	
Extent of coronary vessel involvement				
Normal	19 (16.1)	131 (14.7)	150 (14.9)	0.013
No significant	18 (15.3)	67 (7.5)	85 (8.4)	
CAD	81 (68.6)	692 (77.8)	773 (76.7)	

**Table 2:** Logistic regression.

Characteristics	OR (95% CI)^*∗*^	OR (95% CI)^*∗∗*^	OR (95% CI)^*∗∗∗*^
Opiate nonaddicted females	—	—	—
Opiate-addicted females	1.81 (1.03–3.15) *P*=0.037	1.06 (0.70–1.62) *P*=0.753	0.59 (0.35–0.98) *P*=0.045
Opiate nonaddicted males	—	—	—
Opiate-addicted males	1.09 (0.63–1.88) *P*=0.753	0.81 (0.57–1.16) *P*=0.266	0.74 (0.47–1.18) *P*=0.216

^*∗*^Normal and no significant. ^*∗∗*^Normal and CAD. ^*∗∗∗*^No significant and CAD.

**Table 3:** Logistic regression.

Characteristics	OR (95% CI)^*∗*^	OR (95% CI)^*∗∗*^	OR (95% CI)^*∗∗∗*^
Opiate nonaddicted diabetics	—	—	—
Opiate-addicted diabetics	1.85 (0.97–3.76) *P*=0.088	0.81 (0.47–1.37) *P*=0.431	0.44 (0.24–0.77) *P*=0.004

^*∗*^Normal and no significant. ^*∗∗*^Normal and CAD. ^*∗∗∗*^No significant and CAD.

## Data Availability

The data including names of the patients used to support the findings of this study are restricted by the Birjand University Ethics Committee in order to protect patient privacy. Data are available from Dr. Nahid Azdaki (nahidazdaki@yahoo.com) for researchers who meet the criteria to access the confidential data.
